# Genomic analyses of the ancestral Manila family of *Mycobacterium tuberculosis*

**DOI:** 10.1371/journal.pone.0175330

**Published:** 2017-04-10

**Authors:** Xuehua Wan, Kent Koster, Lishi Qian, Edward Desmond, Richard Brostrom, Shaobin Hou, James T. Douglas

**Affiliations:** 1 Advanced Studies in Genomics, Proteomics and Bioinformatics, University of Hawaii, Honolulu, Hawaii, United States of America; 2 Department of Microbiology, University of Hawaii, Honolulu, Hawaii, United States of America; 3 Microbial Diseases Laboratory, California Department of Public Health, Richmond, California, United States of America; 4 TB Control Program, Hawaii State Department of Health, Honolulu, Hawaii, United States of America; National Renewable Energy Laboratory, UNITED STATES

## Abstract

With its airborne transmission and prolonged latency period, *Mycobacterium tuberculosis* spreads worldwide as one of the most successful bacterial pathogens and continues to kill millions of people every year. *M*. *tuberculosis* lineage 1 is inferred to originate ancestrally based on the presence of the 52-bp TbD1 sequence and analysis of single nucleotide polymorphisms. Previously, we briefly reported the complete genome sequencing of *M*. *tuberculosis* strains 96121 and 96075, which belong to the ancient Manila family and modern Beijing family respectively. Here we present the comprehensive genomic analyses of the Manila family in lineage 1 compared to complete genomes in lineages 2–4. Principal component analysis of the presence and absence of CRISPR spacers suggests that Manila isolate 96121 is distinctly distant from lineages 2–4. We further identify a truncated *whiB5* gene and a putative operon consisting of genes encoding a putative serine/threonine kinase PknH and a putative ABC transporter, which are only found in the genomes of Manila family isolates. Six single nucleotide polymorphisms are uniquely conserved in 38 Manila strains. Moreover, when compared to *M*. *tuberculosis* H37Rv, 59 proteins are under positive selection in Manila family isolate 96121 but not in Beijing family isolate 96075. The unique features further serve as biomarkers for Manila strains and may shed light on the limited transmission of this ancestral lineage outside of its Filipino host population.

## Introduction

The global epidemic tuberculosis (TB) accounts for millions of deaths worldwide every year, and it has been recognized as a World Health Organization (WHO) emergency since 1993 [1]. One-third of the world population is latently infected by tuberculosis-causing bacteria [2]. One major cause of death among human immunodeficiency virus (HIV) carrying populations is TB, and more than 10% of TB cases are associated with HIV [1]. In addition, multidrug-resistant TB (MDR-TB) and extensively drug-resistant TB (XDR-TB) occur globally. Thus, identification of novel biomarkers of global TB-causing bacteria is needed for improving clinical detection and developing new treatments [3].

*Mycobacterium tuberculosis* is one pathogenic bacterial species in the *Mycobacterium tuberculosis* complex (MTBC). Seven human-adapted MTBC lineages are characterized based on the phylogenetic analysis; lineages 1–4 and 7 are *M*. *tuberculosis* strains, and lineages 5 and 6 are *Mycobacterium africanum* [4, 5]. Lineages 1, 5 and 6 are classified as ancient lineages due to the presence of a 52-bp region named TbD1, which is also identified in the cattle TB-causing bacterium *Mycobacterium bovis*, while the remaining lineages are classified as modern lineages due to the absence of TbD1 [6]. SNPs (single nucleotide polymorphisms) based phylogenetic analysis further supports this scenario and lineage 1 is estimated to diverge ~ 67,000 years ago [4, 5]. In addition, modern lineages are more successful than those ancient ones when compared in virulence and geographical spread [7].

The Manila family of *M*. *tuberculosis* was originally defined by investigating IS*6110* polymorphism, spoligotyping, and three gene loci (*oxyR*, *gyrA*, and *katG*) in 48 *M*. *tuberculosis* strains, isolated from patients living in Manila, Republic of the Philippines [8]. Based on geographical distribution and our unpublished data, the Manila family belongs to lineage 1which includes *M*. *tuberculosis* strains circulating in the Philippines and around the rim of the Indian Ocean. In our previous work, we performed whole genome sequencing, *de novo* assembly, and gap closing of two drug sensitive *M*. *tuberculosis* strains from the Manila and Beijing families respectively [9]. However, the Manila family has not been fully characterized at the complete genome level. Here we present comparative analyses of the complete genomes of *M*. *tuberculosis* Manila family isolate 96121, strains in lineages 2–4, and two outgroup strains including *M*. *bovis* and *Mycobacterium canettii*. We also investigate whether the unique features in Manila family isolate 96121 are prevalent in 38 draft genomes of the Manila family. Our findings provide evidence to reveal the unique evolution of this ancestrally derived family and may shed light on the limited transmission of this family of *M*. *tuberculosis*.

## Materials and methods

### Genome sequencing, assembly, and data collection

Whole genome shotgun sequencing of *M*. *tuberculosis* Manila family isolate 11L4601 was carried out on the Ion Torrent PGM platform (Thermo Fisher Scientific, USA). The complete genome sequence of Manila family isolate 96121 was used as the reference genome to assemble PGM reads using 454 gsMapper software (Roche). This Whole Genome Shotgun project has been deposited at DDBJ/ENA/GenBank under the accession LSFJ00000000 (Table A in S1 File). The version described in this paper is version LSFJ01000000. MUMmer 3 package was used for whole genome alignments [10]. The Illumina reads sequenced for 37 Manila strains were downloaded from NCBI database (Table A in S1 File). Reads were trimmed using the ea-utils program [11] and then assembled using the reference assembly method as above. Additional genome sequences were downloaded from the NCBI FTP site as described in Table 1 and Table A in S1 File.

**Table 1:** Strain information list.

Strain name	Accession number	Re-annotated protein coding gene number	Isolated place	TbD1
UT205	NC_016934	4109	Colombia, South America	-
Erdman_ATCC35801	NC_020559	4085	Trudeau Mycobacterial Culture Collection	-
Beijing_96075	NZ_CP009426	4054	Beijing, China	-
Manila_96121	NZ_CP009427	4088	Manila, Philippines	+
CCDC5180	NC_017522	4091	Beijing, China	-
RGTB327	NC_017026	4689	Kerala, South India	-
CTRI-2	NC_017524	4057	Russia	-
NITR204	NC_021193	5237	Tamil Nadu, South India	-
CDC1551	NC_002755	4067	Kentucky/Tennessee, USA	-
KZN605	NC_018078	4064	KwaZulu-Natal, South Africa	-
CCDC5079	NC_017523	4176	Beijing, China	-
KZN4207	NC_016768	4051	KwaZulu-Natal, South Africa	-
KZN1435	NC_012943	4064	KwaZulu-Natal, South Africa	-
NITR202	NC_021192	-	Tamil Nadu, South India	-
RGTB423	NC_017528	4793	Kerala, South India	-
NITR206	NC_021194	4137	Tamil Nadu, South India	-
H37Rv	NC_000962	4069	virulent lab strain	-
H37Ra	NC_009525	4079	attenuated lab strain	-
NITR203	NC_021054	4158	Beijing, China	-
F11	NC_009565	4068	South Africa	-
7199–99	NC_020089	4061	Europe	-

### Clustering of CRISPR (Clustered Regularly Interspaced Short Palindromic Repeats) spacers

CRISPR regions and spacers were identified using CRISPRFinder [12, 13]. A total of 716 spacer sequences from 21 complete genomes of *M*. *tuberculosis* were loaded for all-vs-all BLASTN-short to identify homologous spacer sequences. 90% and 95% were set as stringent cut-off values of minimum aligned length and sequence identity. The MCL program [14] was used to cluster homologous spacer sequences and identify 50 groups. The accumulation curve of spacers and principal component analysis were analyzed and visualized with R package.

### Clustering of ortholog groups

Ortholog groups of proteins were identified using orthoMCL [15] with several additional steps to filter all-vs-all BLASTP results [16]. If the aligned length was less than 80% of the longer length of two protein sequences, the BLASTP result was filtered out. Then if the aligned length was equal to or above 150 amino acids, the BLASTP result with pairwise identity below 30% was filtered out; if the aligned length was less than 150 amino acids, the BLASTP result was filtered by the formula (100 × (0.06 + 4.8 × L^(-0.32 × (1+ e^(-L/1000))))), in which L stands for the aligned length [16]. The pipeline Prokka 1.11 [17] was used to re-annotate genomes and then the pan-genome was further analyzed using the Roary pipeline [18]. Gene accumulation curves were plotted using R package vegan or ggplot2 [19, 20].

### Analysis of SNPs and synonymous/nonsynonymous substitution rates

Core-SNPs were identified using the kSNP v2.13 package [21]. Indels and SNPs were also identified using MUMer 3 package [10]. Synonymous substitution rate (Ks) and the ratio of nonsynonymous substitution rate to synonymous substitution rate (Ka/Ks ratio) were calculated by ParaAT and KaKs_Calculator packages [22, 23]. Density plots of Ka/Ks and Ks values were created using R commands and heat scatter plots of Ka and Ks values were generated by LSD package [24].

### Functional annotation

RNA families were annotated using Rfam database [25]. The secondary structures of RNA sequences were calculated and predicted using the stand-alone ViennaRNA package [26]. The RNA structures were visualized using VARNA 3.92 [27].

## Results

### Genome sequencing, data collection, and assembly

We carried out whole genome shotgun sequencing of *M*. *tuberculosis* Manila family isolate 11L4601, which was collected from a female patient living in the Commonwealth of the Northern Mariana Islands in 2010. We generated 116.5 Mb and the assembly totaled 4,356,842 bases, covering 98.8% of the genome of Manila family isolate 96121 (Table B in S1 File). The assembled contigs aligned well with the genome sequence of Manila family 96121, only yielding random simple repeats located away from the plotted diagonal as seen in Fig A in S1 File. To obtain population information of genomes of Manila family, we downloaded Illumina reads sequenced for 37 Manila strains, which were mapped to the genome sequence of H37Rv strain for SNP-based subgrouping of Manila strains [28]. We trimmed the low-quality ends of Illumina reads using Phred quality score Q30 and performed reference assembly to obtain the 37 draft genomes of Manila strains provided in Table B in S1 File.

For comparative genomic analyses to unveil unique signatures of ancestral Manila family, 19 complete genomes of *M*. *tuberculosis* strains were downloaded from the NCBI database (Table 1). By BLASTN analysis, Manila family 96121 was the only complete genome containing the 52-bp TbD1 region which designated it as an ancestral strain, while the other 20 complete genomes only contained a partial region (~ 27 bp) of TbD1 which designated them as modern strains [6]. All the 38 draft genomes of Manila family strains contained 52-bp TbD1 region.

### Gain and loss pattern of CRISPR spacers revealed that the Manila family was distinctly different from modern strains

**Fig 1:**
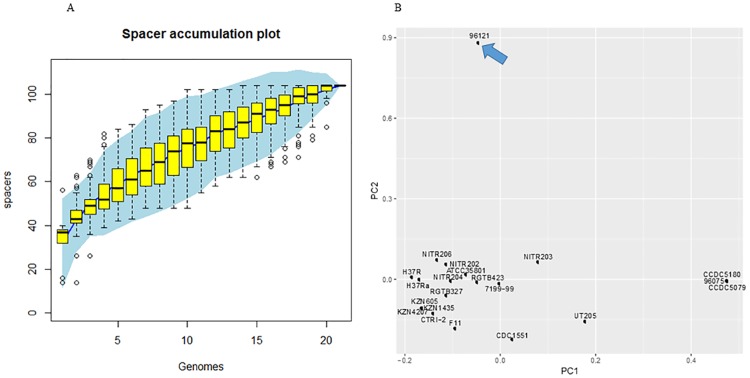
Evolution of CRISPR spacers in complete genomes of 21 *M*. *tuberculosis* strains. (A) Accumulation curve of spacers in complete genomes of 21 *M*. *tuberculosis* strains. (B) PCA of presence and absence of CRISPR spacers in complete genomes of 21 *M*. *tuberculosis* strains. The blue arrow indicates the position of Manila family 96121, illustrating a dramatic difference in the composition of spacers in Manila family 96121.

CRISPR is a repetitive region identified in bacterial and archaeal genomes, and it is proposed as immune system resistant to horizontal gene transfer (HGT) in prokaryotes [29, 30]. We used CRISPRFinder to identify CRISPR regions in 21 complete genomes of *M*. *tuberculosis* and 2 genomes of outgroup strains. The number of CRISPR arrays in *M*. *tuberculosis* genomes ranged from 1 to 3. Two CRISPR regions were identified in Manila family isolate 96121, and one CRISPR region was found in Beijing family isolate 96075. The presence and absence of homologous spacer sequences of each strain were counted from 50 clustered groups and recorded in a binary matrix. The spacer accumulation curve of 21 strains indicated that the pan-genome spacer number for 21 strains was 104 and there was a potentially larger pan-genome spacer size (Fig 1A). Using the Eigenvector method, principal component analysis (PCA) indicated that Manila family isolate 96121 was obviously different from the other 20 strains (Fig 1B). Adding CRISPR spacers of Manila family 11L4601, T17, T92, and outgroup species for PCA, we found that Manila family 11L4601 located closely to Manila family 96121. Furthermore, T17, T92, and *M*. *bovis* located at points between Manila family 96121 and lineages 2–4 (Fig B in S1 File).

**Fig 2:**
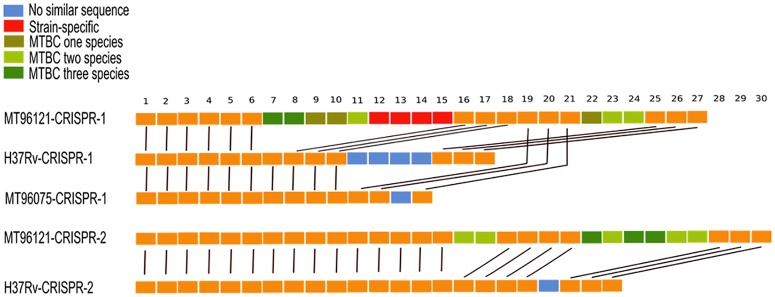
CRISPR spacer rearrangements in Manila family 96121, Beijing family 96075, and H37Rv. Green highlights spacers conserved with other MTBC species but lost in modern Beijing family isolate 96075 and H37Rv. Red highlights spacers specific in Manila family isolate 96121. Blue highlights spacers present in H37Rv but absent in Manila family isolate 96121 and Beijing family isolate 96075.

We further compared spacer region rearrangements among Manila family isolate 96121, Beijing family isolate 96075, and H37Rv to reveal the spacer birth-death pattern of *M*. *tuberculosis* strains isolated from various niches (Fig 2). CRISPR array 1 in Manila family 96121 lacked the acquisition of five spacers in H37Rv, of which the acquisition of spacer 7 occurred in Beijing family isolate 96075. However, it maintained eight homologous spacers with high similarity to those of other species in MTBC including *M*. *bovis*, *Mycobacterium microti*, and *M*. *africanum*. Four spacers in CRISPR array 1 of Manila family isolate 96121 were strain-specific. Comparison of CRISPR array 2 between Manila family isolate 96121 and H37Rv also suggested that eight spacers shared with MTBC were maintained in Manila family 96121 but absent from H37Rv.

### Core-genome and pan-genome analysis of complete genomes revealed genetic diversity in the Manila family

We only considered complete genomes of *M*. *tuberculosis* for core and pan-genome analysis in this study. A total of 82,395 protein sequences from 21 complete genomes of *M*. *tuberculosis* were loaded for all-vs-all BLASTP analysis to identify homologous protein sequences. OrthoMCL [15] was further used to cluster 4,899 groups among strains. Only 736 ortholog groups were identified to contain core genes across the 21 complete genomes of strains, of which 711 orthologous groups contained single-copy orthologous genes from each strain.

**Fig 3:**
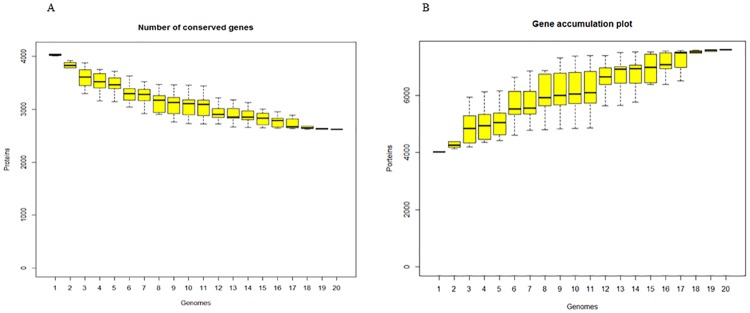
Core-genome and pan-genome of *M*. *tuberculosis* in lineages 1–4, based on analysis of 20 completed genomes. (A) Accumulation curve for conserved genes in 20 re-annotated genomes. (B) Accumulation curve for total gene numbers in 20 re-annotated genomes.

Considering extremely high identities of genome nucleotides of *M*. *tuberculosis* strains, we further questioned whether various gene prediction algorithms caused artificial genetic diversity or whether indels/mutations caused frameshift, premature stop codon, or stop codon readthrough. We used the stand-alone Prokka program [17] to re-annotate 20 complete genome sequences (Table 1), excluding NITR202 strain due to unexpected nucleotide designations. Based on Roary results [18], the core genome included 2,623 orthologous genes and the pan-genome included 7,591 genes (Fig 3A and 3B), indicating that different annotation processes affected core and pan-genome analysis and that considerable genetic diversity within *M*. *tuberculosis* existed. 264 genes in Manila family isolate 96121 were not clustered with genes in H37Rv by Roary. Furthermore, 58 genes in Manila family isolate 96121 were not clustered with genes in 19 re-annotated genomes (Table 1), in which 46 genes were annotated as hypothetical proteins and 12 genes were assigned with functions. We further retrieved coding DNA sequences (CDS) of the 58 genes and ran BLASTN against H37Rv genome sequence to double check whether they were singletons. We manually confirmed that 13 singletons were formed by frameshift or stop codon formation/readthrough and 2 singletons were absent in the 19 complete genomes.

Comparing the genome sequence of Manila isolate 96121 to H37Rv, we identified 983 indels and 2,155 SNPs. 83 genes in the Manila isolate 96121 were identified to contain indels. We suggest that though genome sequences are relatively conserved with high identity, encoded functional proteomes are re-shaped by indels and SNPs, and these microevolutionary changes result in the dynamics and diversity of mycobacterial genomes.

### A truncated WhiB5 protein in the Manila family

We identified one C to T mutation in Manila family isolate 96121 that caused the formation of the stop codon TAG in the *whiB5* gene. This C to T mutation was also present in 38 draft genomes of Manila family, leading to a truncated N-terminal sensor domain that contained four conserved cysteine residues to coordinate the Fe-S cluster but lost the C-terminal alpha helixes that may function to bind DNA or interact with other proteins. The WhiB5 protein in H37Rv is required for expression of 58 genes and is required for progressive and chronic mouse infections [31]. The full CDS of the *whiB5* gene was present in *M*. *canettii*, *M*. *bovis*, and the other 20 *M*. *tuberculosis* complete genomes by BLASTN searches.

### Coupled ancestral ABC transporter and STPK PknH in the Manila family

We identified a unique MTM_01342 gene in the complete genome of Manila family isolate 96121, which was absent from 19 complete genomes of modern strains. MTM_01342 gene was the paralog of MTM_01852 gene, which encoded the forkhead-associated (FHA) domain containing ATP-binding cassette (ABC) transporter in the Manila family isolate 96121 (Table C in S1 File). The CDS of MTM_01342 had no homologous DNA sequence in 19 complete genomes of *M*. *tuberculosis* by BLASTN searches. In contrast, *M*. *canettii* contained the homologous CDS of MTM_01342 gene with 99% identity. We further confirmed that the homologous CDS of MTM_01342 gene was present in 37 Manila strains with 100% identity and that its absence in one Manila strain was due to the fragmented assembly of the draft genome. The above data suggest the loss of the ortholog of putative MTM_01342 gene in modern strains.

In H37Rv, Rv1747 (the ortholog of MTM_01852), the FHA domain-containing ABC transporter, is phosphorylated by Ser/Thr protein kinase (STPK) PknF, and its encoding gene, *Rv1747*, locates adjacent to *pknF* in the genome [32–34]. In the genome of Manila family isolate 96121, MTM_01342 located at ~ 561 kb upstream of MTM_01852, and it was flanked by two *pknH* genes (Table C in S1 File). Coupling the loss of the ortholog of MTM_01342, the paralogous *pknH* gene (MTM_01343) was also absent in most of the modern strains except NITR203 and RGTB423. The intergenic region between MTM_01342 and MTM_01343 contained only 10 bases, suggesting that these two genes may be co-transcribed and form an operon. STPK PknH was reported to phosphorylate the FHA domain of EmbR and helix α10 of DosR, both of which are transcriptional regulators [35, 36]. Deletion of the *pknH* gene in *M*. *tuberculosis* caused hypervirulence in BALB/c mice [37]. Taken together, the genomic locations suggest that the putative FHA domain-containing ABC transporter may be phosphorylated by the putative STPK PknH, and they may function as an additional independent signaling transducer and receptor to regulate virulence in the Manila family.

### SNPs identified in functional coding regions

To investigate SNPs in *M*. *tuberculosis* strains, we used the kSNP v2.13 package [21] to identify SNPs in 21 complete genomes of *M*. *tuberculosis* and 7 draft genomes of Beijing family strains, using the k-mer counting analysis method. After optimization, 11-mers were chosen and generated for SNPs analysis. 158 core SNPs and 2386 non-core SNPs were identified through 28 genomes. Out of 158 core SNPs, 118 SNPs were annotated to be located in protein coding regions of H37Rv, and an additional 17 SNPs were annotated to be located in protein coding regions of strains other than H37Rv. Searching against H37Rv annotation in intergenic regions, we found only two SNPs with functions. To explore SNPs in RNA families, we further annotated 28 genomes with the Rfam database [25]. SNPs in a cobalamin riboswitch and the ASpks small RNA were identified in Manila family isolate 96121. We further used the stand-alone RNAfold program in ViennaRNA package [26] to predict the SNPs’ effects on RNA structure folding. A single-base G to A mutation in the cobalamin riboswitch of Manila family isolate 96121 may cause a local confirmation change (Fig C in S1 File). It was also found in Indian strains RGTB423 and NITR206. A single-base G to C mutation in the ASpks small RNA of Manila family isolate 96121 may cause a global confirmation change, causing a double stem-loop structure to form a single stem-loop (Fig C in S1 File). Again, this mutation was also in Indian strain RGTB423 and NITR206. ASpks was identified as an antisense regulator of pks12 mRNA, which is involved in polyketide biosynthesis and pathogenesis [38].

Compared to H37Rv, 29 out of 158 core SNPs were identified in Manila strain 96121, and 20 SNPs were located in protein coding regions of H37Rv. 8 SNPs were synonymous mutations, while 12 SNPs were nonsynonymous. Annotated functional genes with these nonsynonymous SNPs included the possible protein exporter SecE, methyltransferase, oxidoreductase, polyketide synthase pks15, and others (Table 2).

**Table 2:** List of Manila 96121 nonsynonymous SNPs in protein coding genes.

SNP ID	H37Rv Location	H37Rv base	H37Rv amino acid	Manila 96121 Location	Manila 96121 base	Manila 96121 amino acid	H37Rv name	H37Rv locus tag
T189	3504418	G	P	3510068	A	L	pflA	Rv3138
T337	4013388	G	M	4014984	A	I	kshB	Rv3571
T444	734116	T	M	732013	C	T	secE1	Rv0638
T530	1689349	G	R	1687692	A	H	Rv1498c	Rv1498c
T678	2569593	T	H	2584176	C	R	Rv2298	Rv2298
T857	3296721	G	G	3305059	C	R	pks15	Rv2947c
T907	15117	C	I	15117	G	M	trpG	Rv0013
T1565	895082	A	F	890641	G	L	Rv0802c	Rv0802c
T1594	885689	C	T	884148	A	K	Rv0791c	Rv0791c
T2042	3299413	G	A	3307751	A	V	fadD22	Rv2948c
T2046	3035533	G	D	3044018	A	N	Rv2723	Rv2723
T2264	1113290	G	E	1108916	C	Q	Rv0996	Rv0996

Six unique SNPs were identified in the complete genome of Manila family isolate 96121 and draft genomes of 38 Manila family isolates. Four SNPs were in protein coding genes, including *pflA*, *kshB*, *Rv2723* (*alx*), and *Rv0925c*, while the other two SNPs were in intergenic regions. These unique SNPs may serve as additional biomarkers for Manila family strains. Interestingly, another SNP in the *treZ* gene was in Manila family isolate 96121 and 6 other (15.8%) Manila strains, but not in the remaining 32 Manila strains.

### Purifying and positive selection

**Fig 4:**
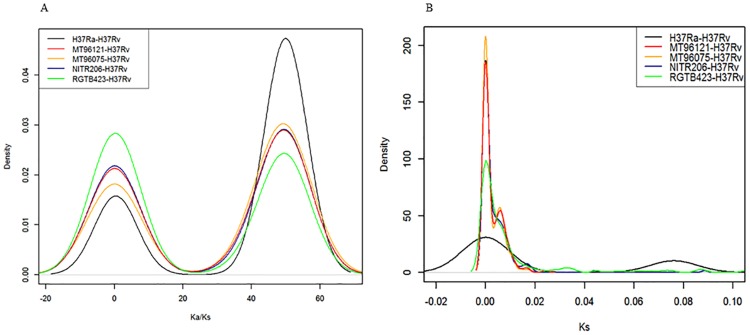
Distributions of Ka/Ks and Ks values for single-copy orthologs among *M*. *tuberculosis* strains. (A) The black, red, yellow, blue, and green lines represent Ka/Ks distributions of single-copy orthologous gene pairs in H37Ra-H37Rv, Manila family isolate 96121-H37Rv, Beijing family isolate 96075-H37Rv, NITR206-H37Rv and RGTB423-H37Rv, respectively. (B) The black, red, yellow, blue, and green lines represent Ks distributions of single-copy orthologous gene pairs in H37Ra-H37Rv, Manila family isolate 96121-H37Rv, Beijing family isolate 96075-H37Rv, NITR206-H37Rv and RGTB423-H37Rv, respectively.

Both purifying selection and positive selection have been reported in *M*. *tuberculosis* genome-wide studies [39–41]. To examine natural selection directions in Manila family isolate 96121 and Beijing family isolate 96075, we scanned 711 single-copy orthologs to calculate and plot densities of Ka/Ks ratios (the ratio of nonsynonymous substitution rate (Ka) to synonymous substitution rate (Ks)), comparing each strain to H37Rv (Fig 4A and 4B, Fig D in S1 File). The densities of Ka/Ks values showed two peaks, one centered at less than 1 and the other centered above 1, indicating that one set of genes are under purifying selection, while the other set of genes are under positive selection. Density plot of the synonymous substitution rate Ks indicated a different pattern for the attenuated strain H37Ra.

A total of 108 proteins were under positive selection in Manila family isolate 96121. 16 proteins were annotated as hypothetical proteins by the NCBI Prokaryotic Genome Annotation Pipeline (PGAP). 59 proteins under positive selection were identified in Manila family isolate 96121 but not in Beijing family isolate 96075 (Table 3). These proteins were encoded by genes involved in diverse functions. These nonsynonymous substitutions may cause changes in protein structure and functional activity. Compared to strain H37Rv, 11 amino acids were mutated from nonpolar amino acids to polar amino acids in Manila family isolate 96121, including the ribosomal protein S9 and the PhoU family transcription/regulation protein.

**Table 3:** List of proteins under positive selection in Manila family 96121 but not Beijing family 96075.

Protein ID	Start	End	Strand	Product
AIQ06604.1	5240	7267	+	DNA gyrase subunit B
AIQ06635.1	39871	41196	-	MFS transporter
AIQ06685.1	92390	93340	+	formate hydrogenlyase
AIQ06723.1	147972	148406	-	F420-dependent protein
AIQ06735.1	160933	161538	+	acetyltransferase
AIQ06768.1	193734	194174	+	cyclase
AIQ06771.1	196926	197723	+	ABC transporter permease
AIQ06915.1	377093	377749	+	hypothetical protein
AIQ06932.1	391858	393207	-	cytochrome P450
AIQ06965.1	438910	440292	+	magnesium transporter
AIQ07080.1	562596	563966	+	membrane protein
AIQ07164.1	643362	644150	+	bromoperoxidase
AIQ07252.1	731634	732119	+	preprotein translocase subunit SecE
AIQ07264.1	745467	746375	+	ROK family transcriptional regulator
AIQ07313.1	795363	796802	+	dehydrogenase
AIQ07411.1	882531	883259	-	transglutaminase-like enzyme
AIQ07419.1	890531	891187	-	succinyl-CoA transferase
AIQ07421.1	893640	894269	+	abortive phage infection protein
AIQ07440.1	909816	911870	-	LytR family transcriptional regulator
AIQ07471.1	944559	945386	-	short-chain dehydrogenase
AIQ07554.1	1036753	1037865	-	phosphate-binding protein
AIQ07573.1	1058781	1059944	+	succinyl-CoA synthetase subunit beta
AIQ07602.1	1092448	1093134	+	transcriptional regulator
AIQ07613.1	1104204	1104797	-	5-formyltetrahydrofolate cyclo-ligase
AIQ07936.1	1459675	1460427	+	ATP synthase F0F1 subunit A
AIQ07961.1	1493984	1496575	+	glycogen phosphorylase
AIQ08030.1	1572174	1572575	-	ribonuclease
AIQ08344.1	1934517	1935977	+	sulfate transporter
AIQ08345.1	1936088	1936951	+	chromosome partitioning protein ParA
AIQ08437.1	2038829	2040049	+	secretion protein EccC
AIQ08693.1	2298175	2299170	+	NAD(P)H nitroreductase
AIQ08698.1	2301793	2302758	-	membrane protein
AIQ08731.1	2350042	2350449	+	hypothetical protein
AIQ08827.1	2449181	2449840	+	conserved lipoprotein LppM
AIQ08912.1	2544083	2544586	+	hypothetical protein
AIQ08916.1	2546103	2546921	-	beta-lactamase
AIQ08928.1	2560636	2561163	+	lipoprotein LppN
AIQ08953.1	2583665	2584636	+	oxidoreductase
AIQ08998.1	2627687	2628454	-	molybdopterin biosynthesis protein moeW
AIQ09020.1	2654411	2654803	+	Fur family transcriptional regulator
AIQ09095.1	2739621	2740154	+	alkyl hydroperoxide reductase
AIQ09342.1	3000837	3001532	-	hypothetical protein
AIQ09443.1	3094683	3095222	-	AsnC family transcriptional regulator
AIQ09460.1	3114882	3115445	-	hypothetical protein
AIQ09573.1	3226665	3227540	+	D-alanyl-D-alanine carboxypeptidase
AIQ09609.1	3306175	3308292	-	acyl-CoA synthetase
AIQ09658.1	3360602	3361612	-	3-isopropylmalate dehydrogenase
AIQ09670.1	3372790	3373974	+	lipoprotein LppZ
AIQ09799.1	3509845	3510933	+	pyruvate formate lyase-activating protein
AIQ09869.1	3585653	3586507	-	aminoglycoside phosphotransferase
AIQ09965.1	3690872	3691537	-	PhoU family transcriptional regulator
AIQ09974.1	3701158	3702057	+	acid phosphatase
AIQ10101.1	3864414	3864869	-	30S ribosomal protein S9
AIQ10155.1	3920827	3921669	-	ABC transporter permease
AIQ10191.1	3979655	3981094	+	hypothetical protein
AIQ10406.1	4199060	4199581	-	tRNA adenosine deaminase
AIQ10452.1	4249217	4250344	+	metal dependent hydrolase
AIQ10466.1	4275887	4277401	+	hypothetical protein
AIQ10528.1	4350498	4351604	+	hypothetical protein

## Discussion

Using comprehensive comparative-genomic analysis methods, we identified genomic features of the *M*. *tuberculosis* Manila family isolate 96121. Our analysis suggested that the pan-genome derived from 20 complete *M*. *tuberculosis* genomes was estimated to contain more than 7000 genes, which is much less than the reported pan-genome of *E*. *coli* [42]. This difference may be due to the longer evolution time of *E*. *coli* strains, which has a more rapid growth rate and has taken tens of millions of years [43] to accumulate mutations, indels, and recombinations, and due to the more diversified ecological niches of *E*. *coli* strains that lead to higher odds of acquisition of xenologs via HGT.

The CRISPR regions were fully conserved in Manila family isolates 96121 and 11L4601, and maintained 16 spacer sequences which were lost in modern lineages but shared high identities with other ancient MTBC species (*M*. *bovis*, *M*. *microti*, and *M*. *africanum*). The international standard method of spoligotyping identifies the presence or absence of CRISPR spacers against a set of preselected oliogos through reverse-line hybridization, but cannot detect mutations or indels. Genomic analysis can identify 57 spacers in the complete genome of Manila family isolate 96121 and show the accurate similarity between spacers from different strains.

Positive selections have been reported in viruses, pathogenic bacteria, and other *M*. *tuberculosis* strains [40, 41, 44]. In this work, the distribution of genome-wide Ka/Ks ratios suggested that one group (75 orthologous genes in Manila 96121-H37Rv) was under purifying selection (Ka/Ks <1) and another group (108 orthologous genes in Manila 96121-H37Rv) was under positive selection (Ka/Ks >1). For decades, synonymous substitution was regarded as silent and neutral and was used to calculate species diversification time. However, recent discoveries suggest that synonymous mutations can cause genetic code bias and affect mRNA stability, and may also be under evolutionary pressure instead of only under neutral evolution [45]. Thus, proteins defined in the purifying selection group may also be the result of natural adaptation.

In this study, we compared Manila family genomes to complete genomes in lineages 2–4 and two outgroup species. We identified the truncated transcription factor *whiB5* gene and an additional potential operon containing the STPK *pknH* and ABC transporter gene. The WhiB5 regulon contains 58 genes, including the alternative sigma factor *sigM* and genes in type VII secretion systems [31]. We hypothesize that the truncated WhiB5 protein may contribute to the Manila family’s limited transmission outside of the Philippines. Gagneux proposed that less virulent MTB strains were selected for during humanity’s hunter–gatherer period, when low population densities meant that more virulent strains would quickly decimate their susceptible hosts, while less virulent strains could be transmitted to a new generation of hosts by reemerging after a period of latency in their current hosts [7]. As human population density increased, more virulent strains could have seen higher transmission and greater spread. Thus, Gagneux proposed that the ancient lineage 1 (including the Manila family) evolved towards lower virulence, while the modern lineages evolved increased virulence. This may partially explain the Manila family’s limited transmission outside of the Philippines. However, more data is still needed to support Gagneux’s suggestion of lower virulence for ancestral MTB lineages. While it has been shown that MTB transmission is more likely to occur in sympatric host populations [46], reduced virulence associated with the Manila family has not been demonstrated. Furthermore, while complete deletion of *whiB5* has been shown to result in attenuated progressive and chronic infections in mice [31], the same study showed that their *whiB5* mutant was unable to resume growth after latency, a characteristic not supported by our data on Manila family latency in humans. From our unpublished data of the 267 TB cases belonging to the Manila family in Hawaii from 2002 to 2013, 249 of the cases were in foreign-born persons. Of those 249 foreign-born cases, 200 of them (80.3%) occurred in patients who had resided in Hawaii for at least two years prior to diagnosis, demonstrating that the Manila family is fully able to cause active disease after a period of latency. Considering about 67,000 ~ 70,000 years of independent evolution of lineage 1 [4, 5], during which the last ice age occurred from ~ 60,000 to 20,000 before present, the C to T mutation in the *whiB5* gene may have been selected during this time by Darwinian selection due to the dramatic decline of human population; later it may have been fixed in the Manila family population by founder effect.

MTM_01342 and MTM_01343 encode the putative FHA domain-containing ABC transporter and STPK PknH separately. MTM_01342 was lost in the modern strains, while MTM_01343 was lost in most of the modern strains except NITR203 and RGTB423. The paralog of MTM_01342 gene in H37Rv, *Rv1747*, is required for *in vivo* growth in mice model but not for *in vitro* growth [32], suggesting that it plays a role in stress response during host-pathogen interactions. We hypothesize that the retention of MTM_01342 gene in the ancestral Manila family may contribute to the host adaptation and limited transmission when Manila family strains compete with the modern strains. Experimental characterization of functions of MTM_01342 will help understand why it is present in Manila family genomes.

Taken together, our findings in this study provide additional biomarkers to identify Manila family strains and may shed light on the limited transmission of this ancestral lineage outside of its Filipino host population.

## Supporting information

S1 File **Table A. Accession numbers of additional strains used in this study. Table B. Summary of genome assemblies of *M*. *tuberculosis* Manila family strains. Table C. Gene list of STPK coupled ABC transporters. Fig A. Alignment of genome sequence of Manila family isolate 11L4601 with 96121 reference sequence**. X axis presents complete genome of Manila family 96121, and y axis presents contigs of Manila family 11L4601. **Fig B. PCA of presence and absence of CRISPR spacers in Manila family 96121 and 11L4601, T17, T92, *M*. *bovis*, *M*. *canettii* and strains in lineages 2–4. Fig C. Predicted secondary structures of cobalamin riboswitch and ASpks small RNA**. (A) Predicted secondary structure of cobalamin riboswitch in Manila family 96121. (B) Predicted secondary structure of cobalamin riboswitch in H37Rv. (C) Predicted secondary structure of ASpks small RNA in Manila family 96121. (D) Predicted secondary structure of ASpks small RNA in H37Rv. **Fig D. Relationship between Ka, Ks, Ka/Ks values in Manila family 96121-H37Rv (A, B, C) and Beijing family 96075-H37Rv (D, E, F)**.
(PDF)
