# Supplementary material for: Genomic analyses of the ancestral Manila family of Mycobacterium tuberculosis
Source: PLoS One. 2017 Apr 10;12(4):e0175330. doi: 10.1371/journal.pone.0175330 (PMC5386241; doi:10.1371/journal.pone.0175330)

Supporting Information – S1 File

Table A. Accession numbers of additional strains used in this study.

Strain name	Accession number
Mycobacterium bovis AF2122/97	NC_002945
Mycobacterium canettii CIPT *140010059*	NC_015848
Mycobacterium tuberculosis 11L4601	LSFJ00000000
Mycobacterium tuberculosis Mtb43	AUPO00000000
Mycobacterium tuberculosis Mtb194	AUNH00000000
Mycobacterium tuberculosis Mtb293	AUPX00000000
Mycobacterium tuberculosis Mtb526	AUTF00000000
Mycobacterium tuberculosis Mtb562	AUTG00000000
Mycobacterium tuberculosis Mtb940	AUTX00000000
Mycobacterium tuberculosis Mtb984	AUTY00000000
Mycobacterium tuberculosis T17	ABQH00000000
Mycobacterium tuberculosis T92	JLDA00000000
Mycobacterium tuberculosis Manila 37 strains	SRP044223

All the sequence data were obtained from RefSeq microbial genomes database [1] or sequence read archive (SRA) [2].

1. **Tatusova T, Ciufo S, Fedorov B, O'Neil K, Tolstoy I**. 2014. RefSeq microbial genomes database: new representation and annotation strategy. Nucleic Acids Res 42:D553-9. doi: 10.1093/nar/gkt1274.

2. **Kodama Y, Shumway M, Leinonen R**. 2012. The sequence read archive: explosive growth of sequencing data. Nucleic Acids Res 40:D54–56. doi: 10.1093/nar/gkr854.

Table B. Summary of genome assemblies of *M. tuberculosis* Manila family strains.

Strain number	Status	Contig number	Contig span (bp)	N50 (bp)	N90 (bp)	SRA or Accession number
Manila_11L4601	Draft	91	4,356,842	114,735	35,954	LSFJ00000000
Manila_1	Draft	109	4,339,014	77,985	27,471	SRR1510036
Manila_2	Draft	110	4,336,215	81,225	22,932	SRR1510037
Manila_3	Draft	114	4,334,586	72,066	25,654	SRR1510038
Manila_4	Draft	120	4,330,802	69,662	22,871	SRR1510039
Manila_5	Draft	110	4,328,695	80,708	24,024	SRR1510040
Manila_6	Draft	99	4,339,983	91,057	31,180	SRR1510041
Manila_7	Draft	103	4,337,723	81,157	27,019	SRR1510042
Manila_8	Draft	101	4,342,152	85,775	33,169	SRR1510043
Manila_9	Draft	100	4,338,984	77,966	27,481	SRR1510044
Manila_10	Draft	106	4,338,449	79,979	25,679	SRR1510045
Manila_11	Draft	94	4,342,689	85,872	36,607	SRR1510046
Manila_12	Draft	106	4,339,835	81,132	27,501	SRR1510047
Manila_13	Draft	115	4,333,470	69,174	22,920	SRR1510048
Manila_14	Draft	116	4,334,365	69,671	24,007	SRR1510049
Manila_15	Draft	116	4,332,963	77,968	23,942	SRR1510050
Manila_16	Draft	95	4,331,941	88,837	27,424	SRR1510051
Manila_17	Draft	105	4,343,605	92,354	27,113	SRR1510052
Manila_18	Draft	114	4,336,931	79,972	22,851	SRR1510053
Manila_19	Draft	107	4,338,582	80,667	26,989	SRR1510054
Manila_20	Draft	103	4,336,813	81,129	25,882	SRR1510055
Manila_21	Draft	115	4,337,005	80,649	24,799	SRR1510056
Manila_22	Draft	117	4,331,814	69,798	22,877	SRR1510057
Manila_23	Draft	116	4,339,381	77,048	24,786	SRR1510058
Manila_24	Draft	116	4,334,008	69,189	25,723	SRR1510059
Manila_25	Draft	101	4,319,731	81,242	26,121	SRR1510060
Manila_26	Draft	97	4,342,432	91,058	28,874	SRR1510061
Manila_27	Draft	98	4,343,878	92,352	31,180	SRR1510062
Manila_28	Draft	117	4,336,092	69,659	24,686	SRR1510063
Manila_29	Draft	102	4,340,936	97,856	29,116	SRR1510064
Manila_30	Draft	120	4,328,792	69,212	20,567	SRR1510065
Manila_31	Draft	111	4,332,699	69,188	25,068	SRR1510066
Manila_32	Draft	107	4,336,126	71,943	27,435	SRR1510067
Manila_33	Draft	112	4,335,608	71,796	23,968	SRR1510068
Manila_34	Draft	102	4,340,033	85,886	24,812	SRR1510069
Manila_35	Draft	106	4,337,735	81,147	26,147	SRR1510070
Manila_36	Draft	114	4,338,365	78,135	24,025	SRR1510071
Manila_37	Draft	114	4,327,777	68,201	23,937	SRR1510072

Table C. Gene list of STPK coupled ABC transporters.

Gene ID	Start	End	Strand	Product
MTM_01341	1408954	1410834	-	Serine/threonine-protein kinase PknH
MTM_01342	1411078	1413474	-	ABC transporter ATP-binding/permease protein
MTM_01343	1413485	1415215	-	Serine/threonine-protein kinase PknH
MTM_01851	1973470	1974900	+	Serine/threonine-protein kinase PknF
MTM_01852	1974968	1977559	+	ABC transporter ATP-binding/permease protein

Fig A. Alignment of genome sequence of Manila family isolate 11L4601 with 96121 reference sequence. X axis presents complete genome of Manila 96121, and y axis presents contigs of Manila 11L4601.

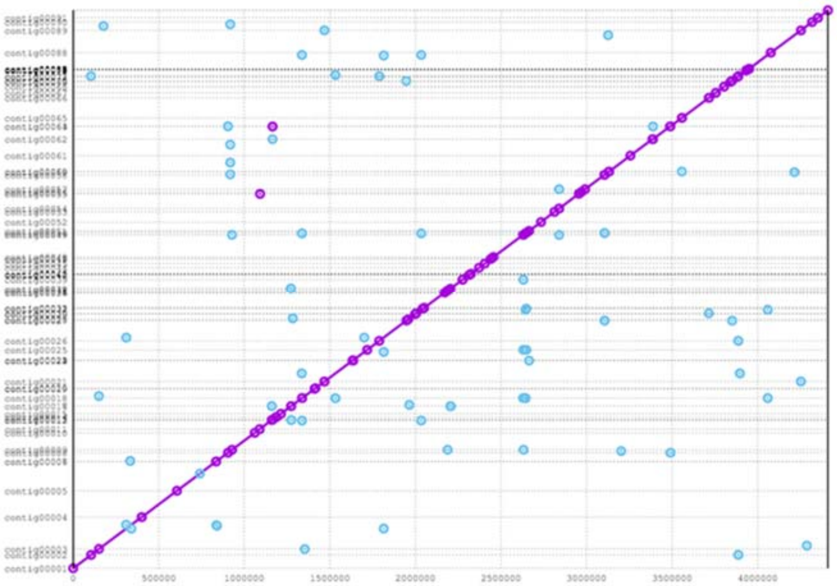

Fig B. PCA of presence and absence of CRISPR spacers in Manila family 96121 and 11L4601, T17, T92, *M. bovis*, *M. canettii* and strains in lineages 2-4.

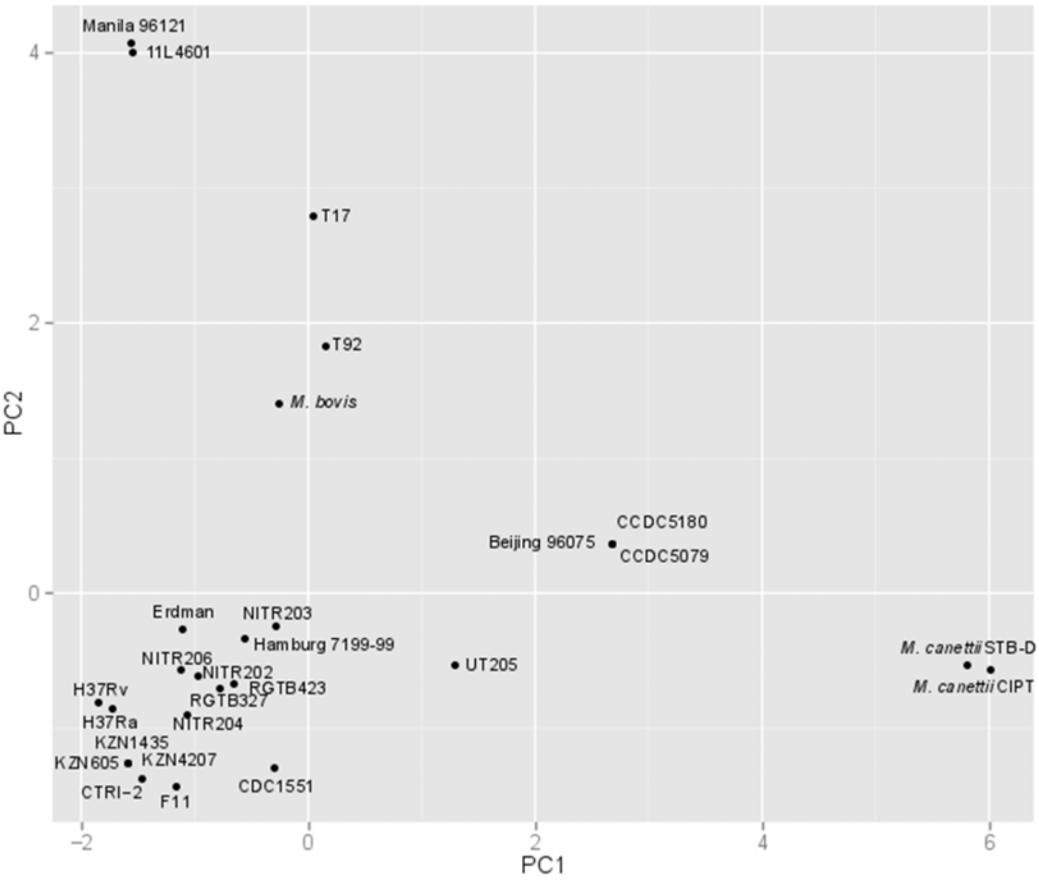

Fig C. Predicted secondary structures of cobalamin riboswitch and ASpks small RNA. (A) Predicted secondary structure of cobalamin riboswitch in Manila family 96121. (B) Predicted secondary structure of cobalamin riboswitch in H37Rv. (C) Predicted secondary structure of ASpks small RNA in Manila family 96121. (D) Predicted secondary structure of ASpks small RNA in H37Rv.

A

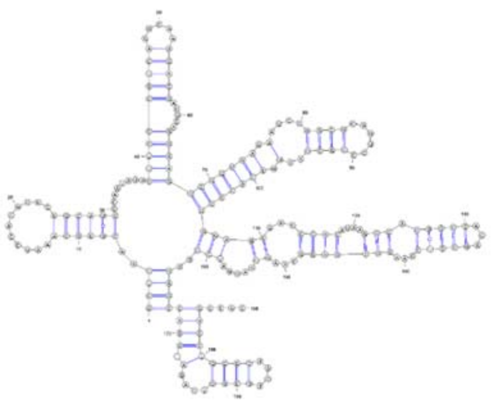

B

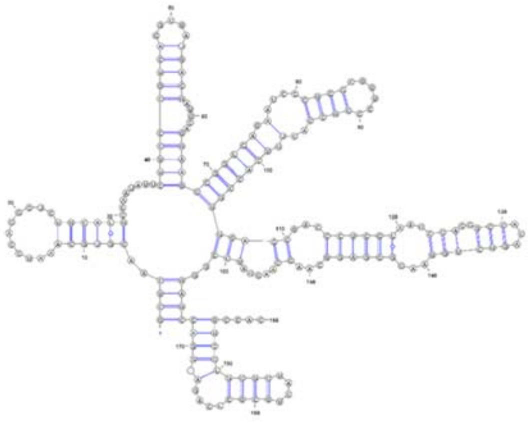

C

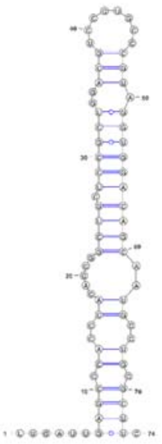

D

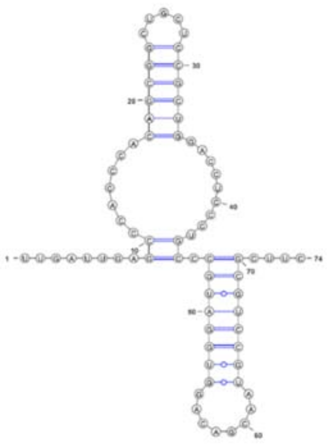

Fig D. Relationship between Ka, Ks, Ka/Ks values in Manila family 96121-H37Rv (A, B, C) and Beijing family 96075-H37Rv (D, E, F).

A

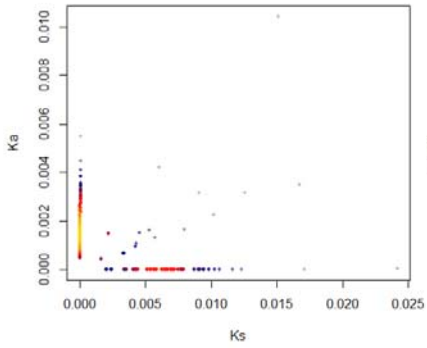

B

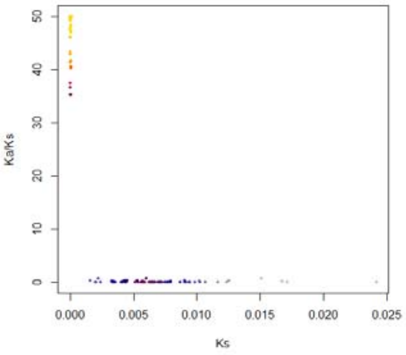

C

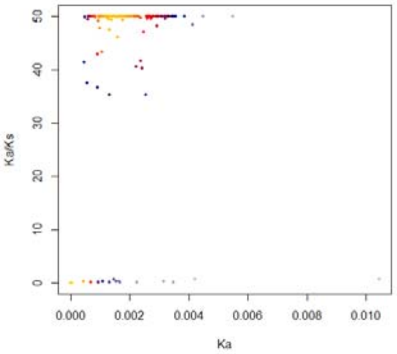

D

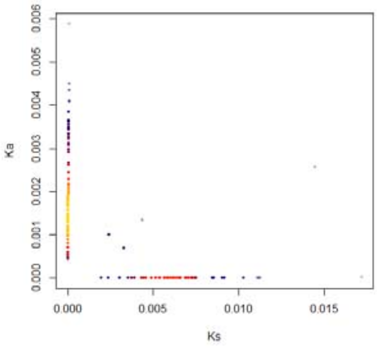

E

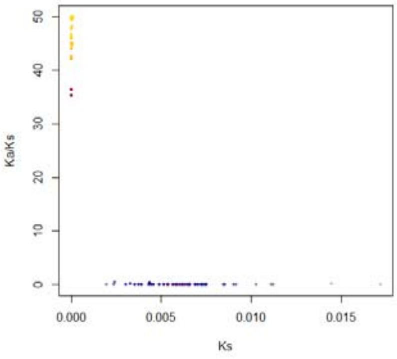

F

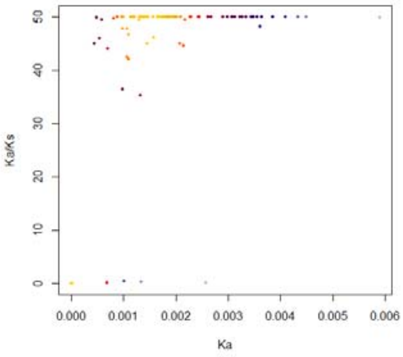

Supplement: S1 File — Table A. Accession numbers of additional strains used in this study. Table B. Summary of genome assemblies of M. tuberculosis Manila family strains. Table C. Gene list of STPK coupled ABC transporters. Fig A. Alignment of genome sequence of Manila family isolate 11L4601 with 96121 reference sequence. X axis presents complete genome of Manila family 96121, and y axis presents contigs of Manila family 11L4601. Fig B. PCA of presence and absence of CRISPR spacers in Manila family 96121 and 11L4601, T17, T92, M. bovis, M. canettii and strains in lineages 2–4. Fig C. Predicted secondary structures of cobalamin riboswitch and ASpks small RNA. (A) Predicted secondary structure of cobalamin riboswitch in Manila family 96121. (B) Predicted secondary structure of cobalamin riboswitch in H37Rv. (C) Predicted secondary structure of ASpks small RNA in Manila family 96121. (D) Predicted secondary structure of ASpks small RNA in H37Rv. Fig D. Relationship between Ka, Ks, Ka/Ks values in Manila family 96121-H37Rv (A, B, C) and Beijing family 96075-H37Rv (D, E, F). (PDF) [file pone.0175330.s001.pdf]
